# VA Problem-Solving Training During COVID-19 for Clinicians of Patients With Complex Comorbidities

**DOI:** 10.1093/geroni/igab046.107

**Published:** 2021-12-17

**Authors:** Sherry Beaudreau, Marcela Otero, Jessica Walker, Christine Gould, Julie Wetherell

**Affiliations:** 1 VA Palo Alto / Stanford University School of Medicine, VA Palo Alto, California, United States; 2 VA Palo Alto, Palo Alto, California, United States; 3 VA Central Office, Salisbury, North Carolina, United States; 4 VA Palo Alto Health Care System, Palo Alto, California, United States; 5 VA San Diego / UCSD, La Jolla Village, California, United States

## Abstract

To address the shortage of mental health providers in geriatrics, VA has implemented clinician training in a VA Problem Solving Training (PST) protocol adapted to the needs of mostly older patients with complex comorbidities. This presentation will summarize PST implementation adaptations during COVID-19, and compare Veteran treatment outcomes before (2019) and during COVID-19 (2020). Sixty-one clinicians attended a workshop and small-group consultation for two training cases. Consultants provided ongoing feedback to program leadership about pandemic-related implementation challenges. Program adaptations during COVID-19 addressed challenges related to delivering treatment by telephone, video, or in-person and recruitment barriers. Veterans in both cohorts (N = 122) had significant reductions in mental health symptoms from baseline to posttreatment in paired t-test comparisons (ps < .01). Flexibilities afforded to clinicians in the training during the pandemic did not diminish the effectiveness of the intervention, thus supporting continued implementation of the training program with added flexibility.

